# High survival of mouse oocytes/embryos after vitrification without permeating cryoprotectants followed by ultra-rapid warming with an IR laser pulse

**DOI:** 10.1038/srep09271

**Published:** 2015-03-19

**Authors:** Bo Jin, Peter Mazur

**Affiliations:** 1 Fundamental and Applied Cryobiology Group, Department of Biochemistry, Cell, and Molecular Biology, The University of Tennessee, Knoxville, TN, 37996-0840, USA

## Abstract

Vitrification is now the main route to the cryopreservation of human and animal oocytes and preimplantation embryos. A central belief is that for success, the cells must be placed in very high concentrations of cryoprotective solutes and must be cooled extremely rapidly. We have shown recently that these beliefs are incorrect. Over 90% of mouse oocytes and embryos survive being cooled relatively slowly even in solutions containing only 1/3^rd^ the normal solute concentrations, *provided that* they are warmed ultra-rapidly at 10^7^°C/min by a laser pulse. Nearly all vitrification solutions contain both permeating and non-permeating solutes, and an important question is whether the former protect because they permeate the cells and promote intracellular vitrification (as is almost universally believed), or because they osmotically withdraw a large fraction of intracellular water prior to cooling. The answer for the mouse system is clearly the latter. When oocytes or embryos are placed in 1 molal concentrations of the impermeable solute sucrose, they osmotically lose ~85% of their cellular water in less than 2 minutes. If the cells are then cooled rapidly to −196°C, nearly 90% remain viable after warming, again provided that the warming is ultra rapid.

*“To survive vitrification, cells must be suspended in and permeated by solutes in the protective solutions in which they are immersed.”* So believe the great majority of cryobiologists. Over the past six years, we ourselves have used variants of a vitrification solution, EAFS^1^, which is also composed of a mixture of permeating and non-permeating solutes. The former are ethylene glycol (EG) and acetamide; the latter are Ficoll, sucrose, and salt. Using a 3-fold dilution of that medium, we have obtained over 90% survival of mouse oocytes provided that after vitrification, the samples are warmed at the ultra-high rate of 1 × 10^7^°C/min by the application of an IR laser pulse^2^. The extremely high rate appears to protect by impeding the recrystallization of small intracellular ice crystals formed during cooling.

But there is a major problem in attributing the protection to the permeation of protective solutes. Cells placed in solutions containing permeating solutes initially shrink rapidly from the osmotic loss of water and then re-expand more slowly as the permeating solutes penetrate and water renters to maintain osmotic equilibrium. The result is the so-called shrink-swell curve. In the case of mouse oocytes, the minimum volume is reached after about 1 min exposure at room temperature^3^ (and K. Edashige, personal communication). That minimum volume represents the balance between the last increment of water leaving the cell and the first increments of permeating solutes entering it. We initiated vitrification at 2.0 minutes, the time which Pedro^4^ found yields the maximum survival after vitrification in EAFS. Since, very little permeating solute can have entered the cell in that additional minute, we hypothesize that its ability to survive vitrification and warming depends more on the fraction of cell water that has been pulled out of the cell prior to initiating cooling than on the moles of cryoprotectant that have entered the cell interior.

The purpose of the study reported here was to examine that hypothesis in another way; namely, by suspending oocytes and various stage embryos in vitrification solutions composed only of the non-permeating solutes sucrose, Ficoll, and PB1 (isotonic phosphate buffered saline). The oocytes were stage MII. The embryos were 2- and 8-cell and morulae.

In previous studies, Seki and Mazur^5^ had achieved a measured warming rate of 117,000°C/min by transferring a Cryotop with 5 oocytes in a 0.1 μl droplet of medium from −196°C into 0.5 M sucrose in isotonic phosphate buffered saline at 23°C. Mazur and Kleinhans conceived the idea that the vitrified or frozen oocytes or embryos could be warmed perhaps 100 × more rapidly by applying a powerful short duration laser pulse to the droplet on the Cryotop. A laser manufactured by LaserStar Corp met our requirements of energy and power with one important exception– it emits at 1064 nm in the infrared but the water-rich medium and cell contents absorb poorly at that wave-length (~3.5%). The resolution to this problem proved to be the introduction of a low concentration of carbon black (India Ink) into the solution. Since carbon black absorbs all wave lengths, it would absorb the laser IR energy and transfer the resulting heat to the surrounding solution, which in turn would transfer it to the oocyte or embryo, The ink particles are too large to penetrate the zona pellucida, the non-living envelope surrounding oocytes and embryos and thus can not come in contact with the plasma membrane. Moreover, the incident laser energy absorbed by the cells themselves is too low to cause injury^6^,^7^.

The vitrification procedure was initiated by placing a 0.1 μl volume of the test solution containing 5–6 oocytes or embryos on the 20 × 0.7 × 0.1 mm blade of a Cryotop, The Cryotop was then placed on a special Cryo-Jig and its blade cooled at 69,000°C/min by immersion in liquid nitrogen. To warm, the blade was abruptly lifted out of the LN_2_, positioned under the laser, and the laser fired within 0.25 s with a pulse duration of 1 ms. This produced a calculated warming rate of 10^7^°C/min between −180°C and −3.5°C. Details are given below and in Ref. 2.

Two methods were used to assess survivals after treatment. One, “morphological survival”, was based on membrane intactness, osmotic responsiveness, and morphological normality^8^. The other was functional survival. For oocytes, that was based on their ability to be fertilized *in vitro* and develop to 2-cell embryos and expanded blastocysts in culture. For embryos, functional survival was based on their ability to develop to expanded blastocysts *in vitro*. Further details are in Methods.

## Results

Table 1 summarizes the morphological and functional survivals of oocytes vitrified in either 0.72 molal sucrose (0×Std-1) or 1.0 molal sucrose (0×Std-2) [Column 1], warmed at 1.2 × 10^5^°C/min (no laser) or 1 × 10^7^°C/min (+ laser) [Column 2]. With the lower warming rate, there were no morphological survivors (Column 4) or functional survivors (Columns 5 and 6). But when the laser was used to warm 100-fold faster, the morphological survivals ranged from 77 to 89% and the 2-cell functional survivals (Column 5) from 78 to 96% of the morphological survival. With laser warming, 61% also developed to expanded blastocysts (Column 6).

The morphological survivals of oocytes vitrified in 1.0 molal sucrose solutions and warmed by laser were slightly but nonsignificantly higher than those vitrified in 0.72 molal solutions (83% and 77%, respectively [p > 0.5]). The mean functional survivals in 1.0 and 0.72 molal sucrose (fertilization and development to 2-cell) were 94% and 78%, respectively, of the morphological survivals, a significant difference (p < 0.05). The 1.0 molal set contained 1-step and 2-step subgroups described in a footnote to Table 1. The mean morphological survivals were 77 and 89%, respectively; the mean 2-cell functional survivals were 91 and 96%, respectively. of the morphological. These differences are not significant (p > 0.05).

The next step was to determine the consequences of vitrification and laser warming on 2-cell and 8-cell embryos and morulae. In these experiments, the mice were naturally mated and the embryos collected after the hours of development required to attain the desired stages. The embryos were then suspended in the 0.72 molal sucrose VS (0×Std-1), vitrified, and warmed with or without a laser pulse. Survival was assessed on a morphological basis or functionally based on their ability to develop to expanded blastocysts. The results are shown in Table 2. Again, none survived either morphologically or functionally when they were warmed at 1.2 × 10^5^°C/min. In contrast, high percentages survived morphologically (Column 4) and functionally (Column 5) after warming 100-times faster with a laser pulse. In the case of the 2-cell and 8-cell embryos, the morphological and functional survivals were 94% and 96% for the two stages, respectively. The morphological and functional survival of the morulae was slightly lower.

**Figure 1:**
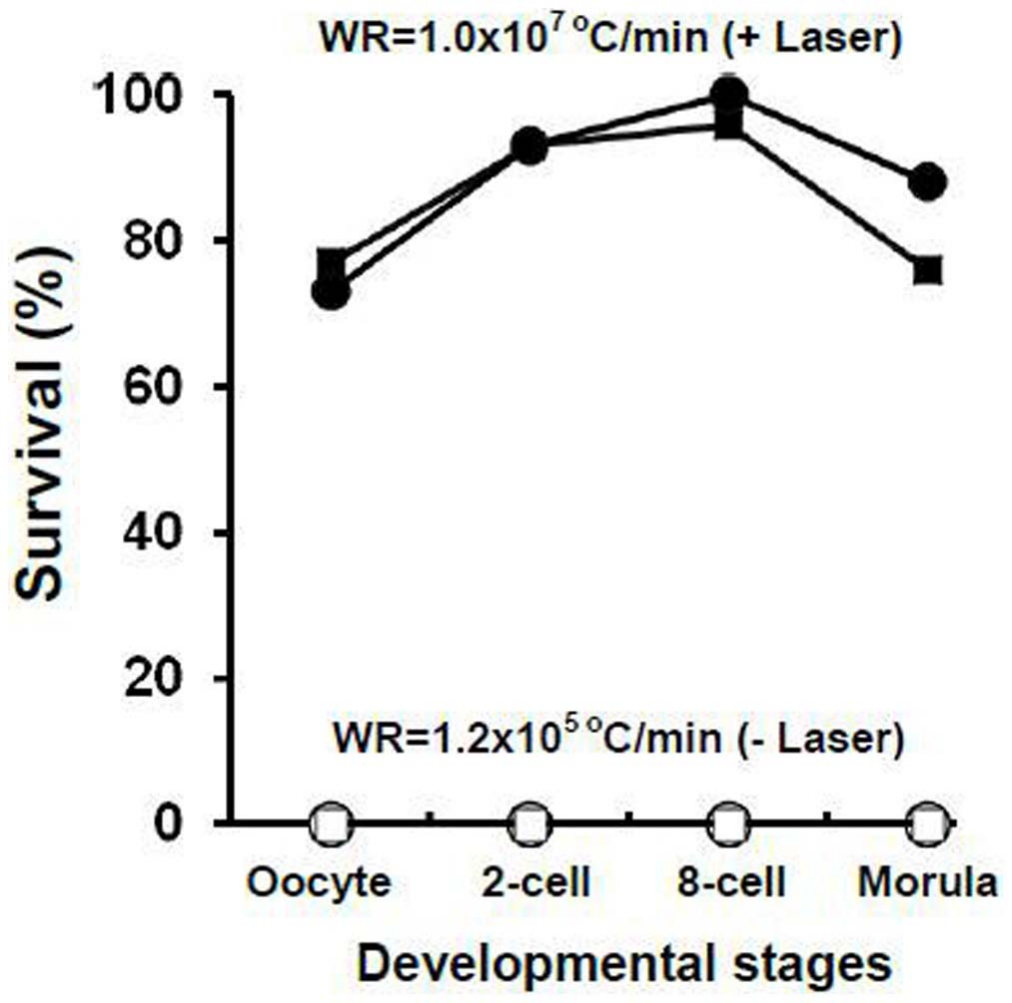
Morphological and functional survival (squares and circles, respectively) of oocytes and embryos at the indicated developmental stages with laser warming (closed symbols) and without (open symbols). WR = warming rate.

These results are summarized in Fig. 1 which plots morphological and functional survival of oocytes and the various stage embryos with and without laser warming. Three conclusions are clear. First, high percentages of oocytes and embryos survived if warmed by laser; none survived if warmed 100 times more slowly. Second, there are few or no differences among the survivals of the various stages, although the 2-cell and 8-cell may possibly survive the best. Third, morphological and functional survivals are very similar.

**Figure 2:**
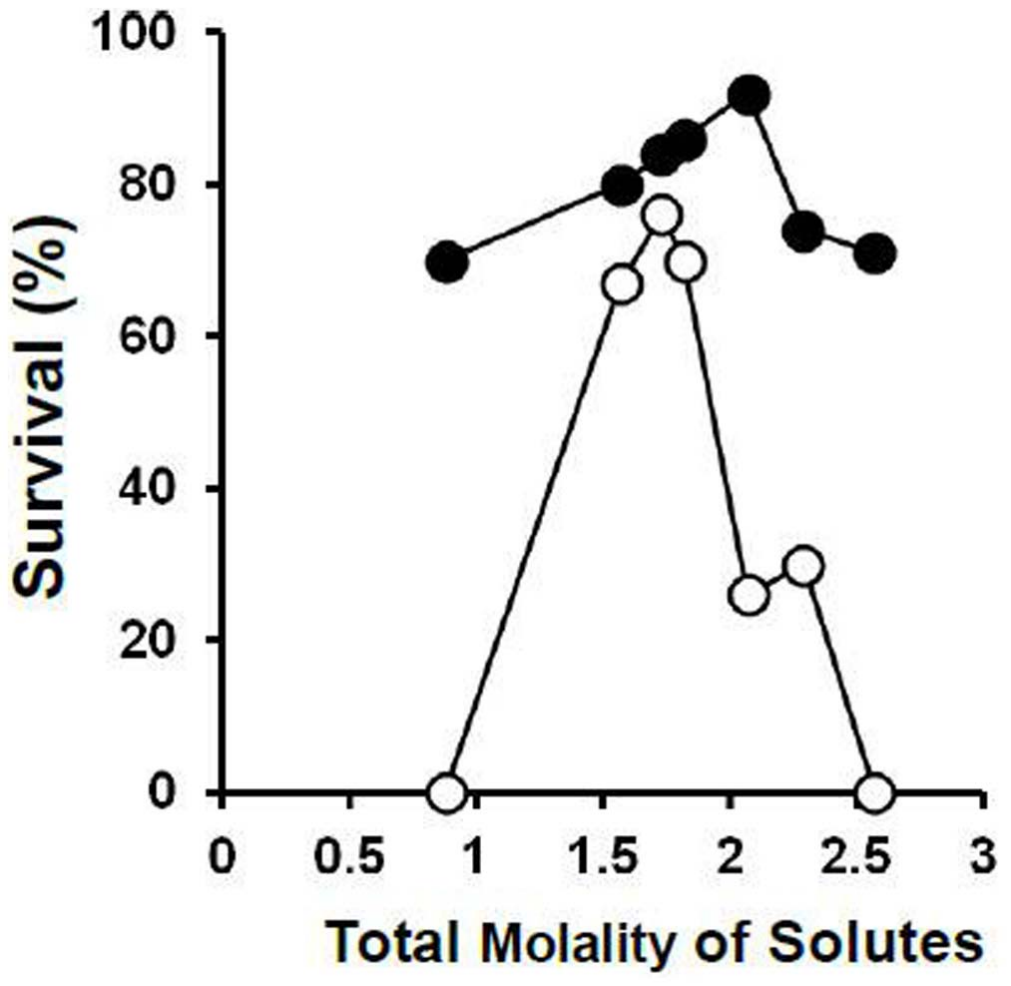
Osmotic/morphological survival of mouse ICR oocytes as function of the total molality of solutes in the modified EAFS solutions in which they were suspended during cooling at 69,000°C/min and warming at 1 × 10^7^°C/min (closed symbols) and 1.2 × 10^5^°C/min (open symbols). The higher rate was by applications of laser pulses [modified from Ref. 2].

**Figure 3:**
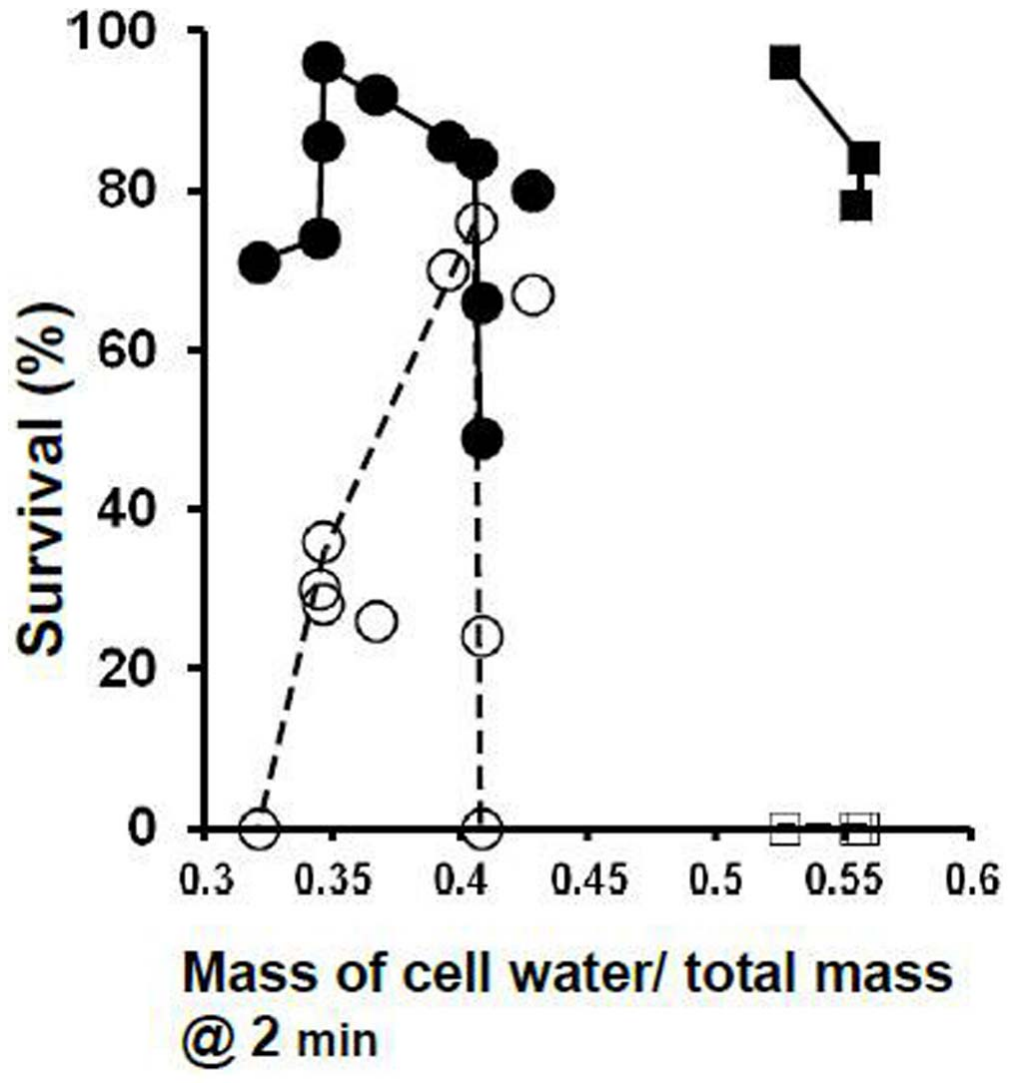
Survival of oocytes with and without laser warming (closed and open symbols, respectively) as a function of the ratio of the mass of cell water to the total mass @2 min. (Left side, circles) Cells suspended in ~0.3 × EAFS-Survivals based on morphology, (Right side, squares) Cells suspended in 0.72 or 1.0 molal sucrose-Functional survivals based on ability to undergo IVF and develop to 2-cell embryos.

The central finding in our original paper on the laser warming of oocytes^2^ was that depicted in Fig. 2; namely, with laser warming, maximum survival (~90%) was obtained when the total molality of the EAFS vitrification solution was around 2 molal. Molalities are moles of solutes per kg of water. The reciprocal of that is related to the mass concentration of water. This can be expressed in several ways, three of which are displayed in Table 3; namely, the mass of water in the oocyte after two minutes of equilibration in the VS as a fraction of the amount in the isotonic cell (Column d), the mass of water in the oocyte after two minutes of equilibration relative to the mass of cell solids (Column f), and the mass of water in the oocyte relative to the total mass of the cell after two minutes of equilibration in the VS (Column g). The survivals listed in the table have been plotted against the third of these in Fig. 3. All three measures of cell water content yield similar shaped curves; the differences being in the numerical values on the abcissa. Panel **a** on the left side of the figure depicts the results for the 0.3 × diluted EAFS solutions in Table 3. The detailed compositions of these solutions is given in Table 1 from Ref. 2. The solid dots and accompanying solid line are the data after laser warming at 1 × 10^7^°C/min. The maximum survival of 96% occurred when the oocytes contained 0.35 g water/total mass of the shrunken cells. But survival remained 80% or higher even when the water content was as high as 0.43 g water/g cells. However, survival decreased abruptly with further increases in water content. Sizeable decreases in survival also occurred with slight decreases in water content below the optimum. When the samples were warmed 100 times more slowly without the laser (open dots and dashed line), this inverted “V” became even sharper. Maximum survival of 76% occurred with a water ratio of 0.41 g water/g but then dropped vertically to zero. It also dropped sharply when the cell water content fell below 0.40 g water/g cells.

## Discussion

Koga^9^ studied various measures of water mobility in yeast cells as a function of their water content expressed as we have done in Columns (d) and (g) in Table 3 and Fig. 3; namely, percent of original water content and the ratio of the mass of cell water to the total mass of the osmotically dehydrated cell. His measures of water mobility were dielectric constant, broad band NMR, differential scanning calorimetry, and adsorption isotherms. All are measures of the mobility of the water molecules and all gave similar results: The water mobility decreases more or less linearly when the yeast cell water content drops below 0.3 g/g total mass and the cell water becomes totally immobilized when its concentration falls to 0.11 to 0.24 g/g. Our data show that the survival of mouse oocytes in diluted EAFS rises to a maximum of 96% as the water content is lowered from 0.43 to 0.35 g/g of osmotically shrunken cells. If mouse oocytes behave like yeast, these values suggest a close correlation between the range in water contents that affect water mobility and the range of water contents that affect survival after vitrification. The connection between the two would presumably be the rapidity at which lethal recrystallization of ice occurs as a function of the cell water content. The more water the cell contains, the faster the warming has to be to thwart recrystallization.

Table 4 and the right side of Fig. 3 depict the very different results for mouse oocytes vitrified in a VS that contain 0.72 or 1 molal sucrose (impermeable) but neither permeating EG or acetamide. Two aspects are prominent. One is that when the samples were warmed ultra-rapidly by laser, close to 90% survived (closed squares) even using a comparatively water-rich VS medium containing 0.53 g water/g total cell mass. The optimum water mass ratio for the diluted EAFS solutions in the left panel is about 0.35 g/g. The second striking feature is that irrespective of their water content, all the oocytes are killed (open squares) when they are warmed 100× more slowly in the absence of the laser pulse.

We currently have no evidence to bear on the detailed physical mechanisms that determine the relation between cellular water content, temperature, and the kinetics of ice recrystallization during warming. Presumably, two important factors are the viscosity of the cell interior and the degree of hydrogen-bonding between the water and intracellular macromolecules as the intracellular water content is reduced. Also still unresolved is why the non-permeating solute sucrose can enhance survival even at molal solute concentrations that are half or less of those required with EAFS?

Probably our most important overall finding is that contrary to the standard belief, the ability to survive vitrification to −196°C and subsequent warming is far more dependent on the oocyte being dehydrated (usually osmotically) to a critical water content prior to vitrification than it is to the type or concentration of cryoprotectants in the cell interior. Of almost equal importance is that survival of dehydrated oocytes is highly dependent on the rate of warming. This suggests that the rate of recrystallization of intracellular ice is highly sensitive to the residual intracellular water content after vitrification. Although we believe that we may be the first to experimentally demonstrate the central role played by cell dehydration and rapid warming in the ability of mouse oocytes to survive vitrification, the concept was proposed by William Rall twenty-seven years ago^10^.

We believe that our core findings will prove applicable to cell and tissue types other than mouse oocytes and embryos. If so. they may force a major conceptual and practical rethinking of the procedures used to ready cells for vitrification. As mentioned, current thinking is that successful vitrification requires that the cells be permeated by high concentrations of cryoprotective solutes beforehand. Paynter *et al.*^3^ found that although it took only 2 min at 10°C for a mouse oocyte to shrink to its minimum volume in 1.5 M EG, it took 30 minutes for it to swell back to 85% of normal volume. Suppose the rate of efflux of water and the rate of influx of protectant molecules of a cell or tissue is only one-hundredth that of the mouse oocyte both because of lower permeability coefficients and lower surface to volume ratios (and important such examples exist), it would dehydrate in 3 hrs but would require 3,000 minutes or 48 hours to achieve say 75% of full rehydration. The odds of the cell or tissue tolerating the former and the ensuing dehydration ought to be higher than that of a cell surviving both dehydration and rehydration.

### Note added in proof

Five days after the acceptance of this paper, a relevant paper by F.W Kleinhans and P. Mazur was accepted by *Cryobiology*^16^. It is currently published on-line with the title “Physical parameters, modeling, and methodological details in using IR laser pulses to warm frozen or vitrified cells ultra-rapidly”. DOI: 1016/j.cryobiol.2015.02.003.

## Methods

### Chemicals

Unless otherwise noted, chemicals were purchased from Sigma (Sigma-Aldrich Co. St. Louis, USA).

### Obtaining mature (MII) mouse oocytes

The sources of the oocytes were ICR female mice (8–12 weeks old; Harlan-Sprague Dawley, USA). They were induced to superovulate with intraperitoneal injections with 0.1 ml of pregnant mare serum gonadotropin (5 IU; eCG) and 48 h later with 0.1 ml (5 IU) of human chorionic gonadotropin (hCG). Thirteen hours after hCG-injection, ovulated unfertilized oocytes were collected from the ampullar region of the oviducts into PB1 medium. The cumulus cells of the collected MII oocytes were removed by exposing them to PB1 containing 0.5 mg/ml of hyaluronidase.

### Collection of Mouse Embryos

Female ICR mice were induced to superovulate with intraperitoneal injections of 5 IU of equine chorionic gonadotropin (eCG) and 5 IU of human chorionic gonadotropin (hCG) given 48 hr apart, and were housed with ICR male mice. At 45 h or 66 hr after the hCG injection, the 2-cell embryos and 8-cell embryos (uncompacted) were flushed from the oviducts of mated animals using PB1 medium. Morulae (compacted) were flushed from the uteri of the mated animals at 78 hr. Embryos with a normal morphology were washed three times with PB1 medium and pooled in fresh PB1 medium in a culture dish under paraffin oil. Procedures for handling the mice were carried out in accordance with Protocol 0911-0713 approved by the Animal Care and Use Committee of the University of Tennessee, Knoxville on July 3, 2013.

### Pre-vitrification, vitrification, and laser warming

The vitrification procedure was initiated by transferring oocytes and embryos at 23°C into three successive 100 μl drops of vitrification solutions composed of 0.72 (0×Std-1) or 1.0 molal (0×Std-2) sucrose and 0.0062 molal Ficoll PM 70 (70,000 daltons) dissolved in PB1. During the ensuing 1 min 45 s, 5 oocytes or embryos in a 0.1 μl drop of the experimental medium were placed on a Cryotop (Kitazato BioPharma. Co., Ltd, Fuji, Japan). The Cryotop was placed in the special Cryo-Jig described below, and 15 s later, rapid cooling to −196°C was initiated. Thus, a total of 2.0 min elapsed from the first exposure to the vitrification solution to the initiation of cooling. As mentioned, this is the exposure time that Pedro^4^ found to yield the best survivals of ICR oocytes after vitrification. This relatively short time also reduced evaporation from the droplet prior to cooling^11^. We have depicted the Cryotop previously^12^.

A Cryo Jig was used to manipulate the Cryotop during rapid cooling in LN_2_ and ultra-rapid warming by a laser pulse. It was developed by F.W. Kleinhans and is depicted in Ref. 2. For cooling, the blade of the Cryotop holding the cells was abruptly immersed in a small LN_2_-filled bath on the Jig. It cooled at a measured rate of 69,000°C/min^10^. Warming was effected by lifting the Cryotop blade out of the LN_2,_ an act that fired the laser in ≤0.25 s. The procedures are detailed in Ref. 2.

### The warming rate by laser

We know of no method to directly measure the warming rate (WR) produced by the laser pulse; consequently, we have assumed that WR = (Δ*T*/Δ*t*). The first step was to decide on the temperature range (Δ*T*) over which the laser pulse is to be applied. We take the starting point to be −180°C based on our measure of the amount the sample warms in air between its removal from LN_2_ at −196°C and the firing of the laser ~0.2 s later. The end point is −3.5°C, the melting point of 0.33× EAFS vitrification solution. The duration of the pulse needed to warm at the desired rate is Δ*t* = Δ*T*/WR. For the desired warming rates of 1.0 × 10^7^°C/min from −180°C to −3.5°C, the pulse duration needed to be 1 ms.

In previous studies Seki and Mazur^5^ had achieved a measured warming rate of 117,000°C/min by transferring a Cryotop with 5 oocytes in a 0.1 μl droplet of medium from −196°C into 0.5 M sucrose in PB1, a modified isotonic phosphate buffered saline, at 23°C. This procedure served as a comparison in the present study.

### Post warming procedure

After warming from −196°C, the Cryotop and its adhering oocytes were held 10-min in 0.5 M sucrose in PB1. They were then transferred into sucrose-free PB1, washed three times in Cook IVF medium (K-RVFE) (Cook Medical, Bloomington, IN), and then groups of five morphologically normal oocytes were transferred into each of several 100 μl drops of the IVF medium that contained a measured concentration of sperm. These pre-collected sperm had been diluted 100-fold initially and their concentration determined by haemocytometer. They were further diluted to produce samples containing 3 × 10^6^ sperm/ml. After incubation for 5 hr in the Cook fertilization medium at 37°C under 5% CO_2_/95% air, fertilized oocytes were transferred after washing from the Cook IVF medium to 100 μl droplets of Cook Cleavage medium and incubated for 5 days under 5% CO_2_/95% air to permit development to expanded blastocysts.

The procedure for embryos was similar. After warming from −196°C and the 10-min exposure to 0.5 M sucrose in PB1, embryos were transferred to sucrose-free PB1 and then to Cook Cleavage medium (Catalog name: K-RVCL), in which they were incubated for 2 hr at 37°C. The percentage exhibiting normal morphology and volumes at this point were scored as survivors.

The functional survival of naturally developed embryos was assessed by their ability to develop into expanded blastocysts during 96 h of culture for 2-cell embryos and 48–72 h of culture for 8-cell embryos and morulae.

### Statistics

Error figures in tables are standard errors (standard deviations of the mean). Tests of significance were carried out by 2-tailed t-tests.

## Author Contributions

B.J. and P.M. planned the experimental goals; B.J. conducted the experiments. Both contributed to the analyses and the writing of the paper, P.M. is the PI of the supporting NIH grant.

## Figures and Tables

**Table 1:** Morphological and functional survival of mouse oocytes after vitrification in 0.72 or 1.0 molal sucrose media

Vitrification sol'n*	Warming rate (°C/min)†	#egg (#runs)	N (% Morpholog. Normal)	N (% 2-cell)	N (% Expanded Blastocyst)
(1)	(2)	(3)	(4)	(5)	(6)
0×Std-1: 0.72 molal sucrose No vitrific	-	40 (8)	40 (100)	35 (88 ± 3.7)	31 (78 ± 4.5)
0×Std-1: 0.72 molal sucrose with vitrific	1.2 × 10^5^	20 (4)	0 (0)	0 (0)	0 (0)
0×Std-1: 0.72 molal sucrose with vitrific	1 × 10^7^	35 (7)	27 (77 ± 5.2)	21 (78 ± 6.5)	19 (70 ± 8.0)
0×Std-2: 1 molal sucrose No vitrific	-	60 (12)	60 (100)	58 (97 ± 2.2)	43 (72 ± 5.2)
** ****		30 (6)	30 (100)	26 (87 ± 6.7)	24 (80 ± 7.3)
0×Std-2: 1 molal sucrose With vitrific	1.2 × 10^5^	20 (4)	0 (0)	0 (0)	0 (0)
** ****	1.2 × 10^5^	20 (4)	0 (0)	0 (0)	0 (0)
0×Std-2: 1 molal sucrose With vitrific	1 × 10^7^	57 (12)	44 (77 ± 3.5)	40 (91 ± 4.2)	25 (57 ± 4.3)
** ****	1 × 10^7^	35 (7)	31 (89 ± 4.0)	30 (96 ± 3.6)	17 (55 ± 7.3)

*Each solution also contained 0.15 molal salt and 0.0062 molal Ficoll.

**Oocytes first suspended in 0×Sd-1 for 2 min and then transferred to 0×Std-2 for 1 min before vitrification.

^†^The warming rate of 1 × 10^7^°C/min was achieved with a laser pulse. The percentages that are morphologically normal are the numbers in Columns 4/the numbers of oocytes in column 3. The numbers and percentages that are functionally viable are Columns 5/4 and 6/4. After warming, the oocytes were exposed to freshly collected sperm diluted to a concentration of 3 × 10^6^/ml with Cook Fertilization medium.

**Table 2:** Morphological and functional survival of 2 and 8 cell embryos and morulae after vitrification in 0.72 molal sucrose and warming with and without a laser pulse

(1)	(2)	(3)	(4)	(5)
Vitrification sol’n	Warming rate (°C/min)	# embryos (# runs)	N (% Morpholog. Normal)	N (% Expanded Blastocyst)
A. 2-cell embryos				
None (Control)	-	30 (6)	30 (100)	29 (97 ± 3.3)
0×Std-1	1.2 × 10^5^	20 (4)	0 (0)	0 (0)
	1 × 10^7^	29 (6)	27 (93 ± 4.9)	25 (93 ± 4.2)
B. Uncompacted 8-cell embryos				
None (Control)	-	25 (5)	25 (100)	24 (96 ± 4.0)
0×Std-1	1.2 × 10^5^	20 (4)	0 (0)	0 (0)
	1 × 10^7^	25 (5)	24 (96 ± 4.0)	24 (100)
C. Compacted Morulae				
None (Control)	-	35 (7)	35 (100)	33 (94 ± 4.0)
0×Std-1	1.2 × 10^5^	20 (4)	0 (0)	0 (0)
	1 × 10^7^	34 (7)	26 (76 ± 2.8)	23 (88 ± 5.7)

The percentages that are morphologically normal are the numbers in Columns 4/the numbers of embryos in column 3. The numbers and percentages that are functionally viable are the numbers in Column 5/numbers in Column 4.

**Table 3:** Survival* after laser warming of mouse oocytes vitrified in 0.3× EAFS solutions as a function of the fraction of cell water at the time of vitrification

(a)	(b)	(c)	(d)	(e)	(f)	(g)		
No.	Solution	Total external molality	***Vw_l_*** *(**~W_w_**) after 2 min*	Rel mass of cell solids	Mass of cell water @ 2′/mass of solids	Mass of cell water/Total mass @ 2 min	% Survival with laser warming (1 × 10^7^°C/min)	Survival w/o laser warming (1.2 × 10^5^°C/min)
	1×Std	7.38	0.040	0.233	0.178			91.7 ± 6.3
1	0.33×Std F0	1.72	0.160	0.233	0.689	0.408	49 ± 8.9	0
2	0.33×Std	1.72	0.160	0.233	0.689	0.408	66 ± 6.0	24 ± 5.0
3	0.33×-1 F0	2.28	0.123	0.233	0.530	0.346	86 ± 4.8	36 ± 10
4	0.33×-1	2.28	0.123	0.233	0.530	0.346	96 ± 2.7	28 ± 8.0
5	0.33×-2	2.29	0.123	0.233	0.528	0.345	74 ± 6.8	30 ± 9.3
6	0.33×-3	2.57	0.110	0.233	0.473	0.321	71 ± 7.3	0
7	0.33×-4	2.07	0.135	0.233	0.580	0.367	92 ± 3.3	26 ± 7.2
8	0.33×-5	1.82	0.152	0.233	0.654	0.395	86 ± 4.7	70 ± 6.5
9	0.33×-6	1.73	0.160	0.233	0.685	0.406	84 ± 7.5	76 ± 7.5
10	0.33×-7	1.57	0.174	0.233	0.749	0.428	80 ± 3.8	67 ± 8.4

*Survivals are from Table 1 in Ref. 2*.* They were based on morphology and osmotic integrity. Functional survivals based on percentages of normal-morphology oocytes developing to 2-cell embryos after IVF were also determined for solutions 7 and 8. They were 83% and 86%, respectively.

Columns (a, b, c) from Table 1 of Jin et al.^2^

Column (d): The volume of water in the cell (Vw) as a fraction of the initial isotonic water volume (approximately equal to the ratio of the water masses) after 2 min osmotic equilibration with the external medium assuming the van't Hoff relation, ***M_iso_/M_ext_ = *** 0.3/(molality of ext solution + 0.15 to account for the fact that NaCl forms 2 ions)*.*

Column (e): The volume fraction of the isotonic zona-free mouse oocyte that is occupied by cell solids is 0.18^13^. Protein constitutes 71% of the dry mass^14^, and typical proteins have a density of 1.36 g/cm^3^; e,g,^15^. Consequently, if we assume this to be the density of all the cell solids, the mass of cell solids relative to the volume of an isotonic oocyte is 0.18*1.36 g/cm^3^ of isotonic cells or 0.233 g/g of cells, assuming the density of the zona-free isotonic oocyte to be 1.05 g/cm^3^.

Column (f): column (d)/0.233; i.e., mass of cell water at 2 min/mass of cell solids.

Column (g): Column (d)/(Column (d) + Column (e)); i.e., mass of cell water at 2 min/total cell mass at 2 min.

**Table 4:** Functional survival* of oocytes vitrified in 0.72 or 1.0 molal sucrose + isotonic PB1 + Ficoll as a function of the fraction of cell water at the time of vitrification

(a)	(b)	(c)	(d)	(e)	(f)	(g)		
No.	Solution	Total external molality	***Vw_l_*** *(**~W_w_**) after 2 min*	Rel mass of cell solids	Mass of cell water @ 2′/mass of solids	Mass of cell water/Total mass @ 2 min	% Survival with laser warming (1 × 10^7^°C/min)	Survival w/o laser warming (1.2 × 10^5^°C/min)
11	0×Std-1	0.88	0.291	0.233	1.25	0.555	78 ± 6.5	0
12	0×Std-1a	0.87	0.294	0.233	1.26	0.558	84 ± 3.5	0
13	0×Std -2	1.156	0.260	0.233	1.12	0.527	96 ± 3.6	0

*Survivals are based on the percentages of morphologically normal oocytes developing to 2-cell embryos after *in vitro* fertilization. Morphological survivals were 77% and 83% in 0.72 and 1.0 molal sucrose, respectively.

Column (c): The solutions contained 0.72 or 1.0 molal sucrose plus 0.15 molal PB1 salts and 0.0013 or 0.0062 molal Ficoll.

See footnotes in Table 3 for the procedures used to calculate Columns (d), (f), and (g).
